# Rhabdomyosarcoma of Urinary Bladder

**Published:** 2014-05-21

**Authors:** Vinod Priyadarshi, Debojit Gogoi, Malay Kumar Bera, Dilip Kumar Pal

**Affiliations:** Department of Urology, Institute of Postgraduate Medical Education and Research, Kolkata. India

**Dear Sir,**

Rhabdomyosarcoma (RMS) is the most common soft tissuesarcoma in infants and children. RMS can occur in any age but approximately 87% of patients are younger than 15 years.Approximately25% of these tumors arise from the genitourinary (GU) tract and another 10% from pelvic or retroperitoneal sites.[1]

A 10 year old boy presented with straining, weak stream, sense of incomplete voiding, suprapubic discomfort and occasional dribbling of urine for 6 months. General and systemic examinations revealed no abnormality. Urine examination showed plenty of red blood cells (RBCs) and few pus cells; however the culture was sterile. Hematological and biochemical investigations were normal.Ultrasonography of abdomen suggested a bladder mass of size 5x3x3 cm arising from the left posterolateral wall and impinging on the bladder neck. Kidneys,ureters, retroperitoneum and pelvic cavity were normal. Contrast enhanced CT (CECT) scanreconfirmed the large heterogeneous bladder mass arising fromleft lateral wall of the urinary bladder. Perivesical fat planes were maintained (Fig. 1). Metastatic workup was negative.Urine cytology did not reveal malignant cells.

**Figure:**
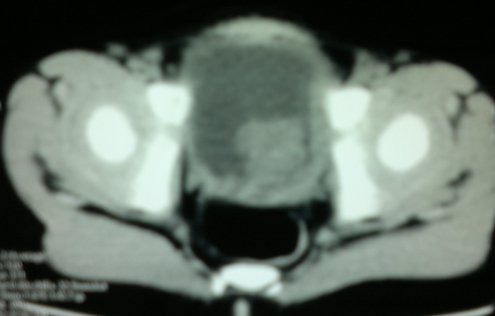
Figure 1: CECT showing large heterogeneous enhancing bladder mass arising from left lateral wall of the urinary bladder.

On cystoscopy a large faint pinkish polypoidal proliferative growth was seen arising from left posterolateral wall that was extending upto the bladder neck and encroaching almost half of bladder lumen(Fig. 2). Left half of the trigone was infiltrated by the mass and left ureteric orifice could not be located.Bladder neck was obliterated by the mass and rest of the bladder was trabeculated. Trans urethral resection of the bladder tumor(TURBT) was done. Histopathological examination showed the presence of elongated cigar-shaped cells with pleomorphic nuclei and skeletal muscle like cross-striations in the cytoplasm, characteristic of rhabdomayoblasts along with abundant population of small,pleomorphic round cells, numerous giant cells and spindle cells; suggestive of RMS(Fig. 3).Partial cystectomy was not feasible due to posterior location of the tumor.Patient was treated with 4 cycles of vincristine and actinomycin D based chemotherapy followed by external beam radiation.Patient remained on regular follow up and became tumor free after 18 months. At 28 months of follow up, there is no evidence of tumor recurrence and patient is doing well.

**Figure:**
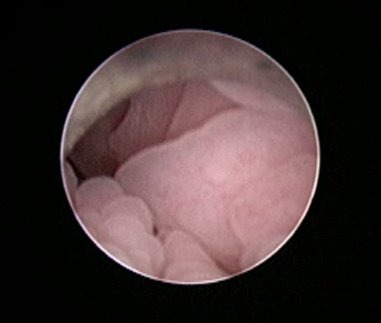
Figure 2: Cystoscopy showing faint polypoidal proliferative growth arising from left posterolateral wall.

**Figure:**
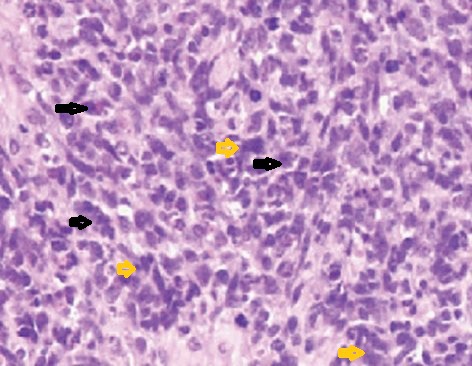
Figure 3: Histopathology showing RMS; black arrowheads-rhabdomayoblasts, yellow arrowheads- giant cells.

Bladder and prostatic primaries account for 50%of genitourinary sarcomas. The tumour originates mainly in the submucosa of posterior bladderwall with particular preference for the regions of bladder neck and trigone [2]. The outcome of children with RMS has improved dramatically over the past 30 years. According to IRS V there are three primary protocols that are based on the risk of disease recurrence with the overall aims of reducing cyclophosphamide and radiation in low-risk groups, evaluating new regimens for unfavourable disease, and applying molecular studies for occult metastasis. The low-risk protocol is recommended for localized embryonal and botryoid histology patients and only vincristine and actinomycin D based chemotherapy is used for node-negative tumors at favorable-site (GU non-bladder/ prostate) and small(≤ 5 cm) node-negative tumors at unfavourable-sites. The three drug VAC regimen is reserved for large(>5 cm) tumor at unfavorable sites with microscopic residual, or positive nodes and for tumor at favorable sites with gross residual or positive nodes.[1] According to IRS V, index patient belongs to low-risk RMS; therefore was treated with two drug chemotherapy regimen (vincristine, actinomycin- D) followed by external beam radiation as the standard treatment [1,3] 

Because of the rarity of tumor, no guideline yet has been set for follow up of bladder RMS. We followed up this case with history, physical examination, urine cytology and ultrasongraphy of abdomen and pelvis (3 monthly),CECT (6 and 12 months and then yearly), and cystoctoscopy(3,6 and then 12 monthly). Though the chance of recurrence is more in first two years, these patients should be followed for long time as delayed recurrence has been reported.

## Footnotes

**Source of Support:** Nil

**Conflict of Interest:** None declared

